# Editorial: Recent advances in rhinology 2024

**DOI:** 10.3389/falgy.2025.1766479

**Published:** 2026-01-07

**Authors:** Diego M. Conti, Glenis K. Scadding

**Affiliations:** 1 Escuela de Doctorado UAM, Centro de Estudios de Posgrado, Universidad Autónoma de Madrid. Calle Francisco Tomás y Valiente, n° 2. Ciudad Universitaria de Cantoblanco, Madrid, Spain; 2 Allergy and Clinical Immunology Research Unit, Department of Microbiology and Immunology, KU Leuven, Leuven, Belgium; 3 Department of Allergy & Rhinology, Royal National ENT Hospital, London, United Kingdom; 4 Division of Immunity and Infection, University College, London, United Kingdom

**Keywords:** allergic rhinitis, biologics, chronic rhinosinusitis, EUFOREA, rhinology, type 2 inflammation

## Introduction

Upper airway diseases, including allergic rhinitis (AR) and chronic rhinosinusitis (CRS), are significant contributors to global respiratory morbidity, manifesting a close association with asthma and other lower-airway disorders (1, 2). Recent advances have highlighted the central role of Type 2 inflammation, epithelial–immune interactions, and environmental exposures in driving disease phenotypes and endotypes (3). Furthermore, recent advances in management have been influenced by precision medicine strategies, including biologics, innovative surgical techniques, and structured patient-reported outcome measures (4).

The articles collected in this Research Topic examine the burden of disease, environmental and immunological interactions, measures to improve airway care, and emerging technological innovations.

## Burden of AR

Jones et al. assessed the burden of AR through an observational epidemiological study. They compared healthcare resource use (HCRU) between patients who were diagnosed in primary care vs. those who had secondary care diagnosis. Most patients (89.3%) received a primary care diagnosis only. Those diagnosed with AR then experienced an increase in mean GP visits [7.5–10.0 per patient per year (PPPY)], respiratory-related hospitalizations (5.5–7.1 PPPY), and AR-related medications (mean 8.8–15.0 PPPY). However asthma incidence was lower after AR diagnosis than before, suggesting that earlier AR diagnosis and management may reduce asthma occurrence. Patients with AR impose a considerable burden on the healthcare system following diagnosis, especially those requiring subsequent respiratory specialist consultations.

## Environment

Investigation of epithelial-immune interactions in allergic disease represents core immunological research that advances our understanding of respiratory tract immunity and the pathogenesis of inflammation (5).

Lal et al. provide novel data that supports the hypothesis that insults to the environment may be an important factor in the pathogenesis of chronic rhinosinusitis (CRS) through epigenetic mechanisms, resulting in dysregulated mRNA transcription and cytokine expression. The multi-omics analysis utilised (unique in its class) demonstrated a clear separation between clusters of control and CRS subjects, thereby validating its use in future research. Furthermore, the investigation revealed interactions between methylated DNA, mRNA, and cytokines in the pathogenesis of CRS, emphasising the identification of novel molecules and pathways that could serve as potential therapeutic targets.

Mucus production in excess is a hallmark of many respiratory diseases, including AR (6). Li et al. sought to evaluate the expression of SerpinB2 on epithelial cells in AR and ascertain whether it regulates MUC5AC via STAT6 signalling. Their study found that there was an increase in nasal epithelial SerpinB2 expression, and that this contributed to MUC5AC expression through the activation of STAT6.Targeting SerpinB2 may offer therapeutic benefits in AR treatment.

## Airway care

Current management of patients with respiratory diseases is suboptimal (7). Several articles in this Research Topic were directed towards measures for improving this situation.

Hellings et al. provide an overview of the European Summit “Raising the bar in respiratory care”, which was organised by EUFOREA at the European Parliament. The objective behind this event was to address the needs of European patients afflicted by chronic respiratory diseases (CRDs) by fostering collaboration with European and global organisations in the domain of CRD management. The event also recognised the limitations of current care models and emphasised the importance of collaborative efforts to enhance care and prevention.

Conti et al. reported on the inaugural European Biologic Training Course, conceived with the objective of addressing the educational needs of physicians engaged in the management of asthma and Chronic Rhinosinusitis with Nasal Polyps (CRSwNP). The course focused on clinically relevant facets of diagnosing these diseases and on using biologics appropriately in their treatment. This included patient selection for biologic drugs, communication, the initiation of treatment and the monitoring of patients in everyday clinical practice.

Conti et al. documented the Workshop, entitled “Primary Care Physicians and Nurses with an Interest in Global Airway Diseases 2024”, which aimed to raise awareness of the global airway concept within the field of medical care. It provided a distinctive perspective on key aspects of respiratory health, including prevention, diagnosis and treatment. A central theme of the programme was the importance of referral to specialist settings and the recognition of secondary care specialists in upper and lower airway diseases. Understanding the repercussions that associated multimorbidities have on patients when their conditions are not adequately treated or managed and acquiring evidence-based counsel for screening and referral to specialist settings were pillars of this workshop.

Scadding et al. addressed the need for fundamental updates to the definitions of AR to enable effective communication among healthcare specialists involved in its treatment and to enhance patient care. This report offers a consensus on updated AR definitions, encompassing control, severe allergic rhinoconjunctivitis, refractory severe allergic rhinoconjunctivitis, remission, resolution, improvement, exacerbation, treatable traits, treat to target, relapse, progression, disease modification, and prevention.

## Chronic rhinosinusitis

Alao et al. emphasise the necessity for original, minimally invasive, and environmentally sustainable therapeutic strategies in the management of chronic airway inflammation. The review explores the domain of acoustic therapy, a relatively novel treatment modality that utilises sound wave-induced vibrations with the aim of providing a complementary therapeutic intervention for AR and CRS. In consideration of the extant, albeit circumscribed, corpus of evidence, a transdisciplinary approach is adopted, incorporating perspectives from the domains of respiratory medicine, immunology, and microbiome science, thereby establishing a translational framework for future research endeavours.

Blauwblomme et al. present a unique illustration of a patient afflicted with CRSwNP, severe eosinophilic asthma, and uncontrolled upper airway symptoms, who demonstrated notable therapeutic benefits through dual monoclonal antibody therapy. The incorporation of an anti-IL-4R*α* antibody resulted in a reduction of upper airway symptoms and a decrease in both eosinophilic and neutrophilic inflammation, irrespective of prior anti-IL-5 therapy. These effects were observed in both polyp tissue and nasal secretions. This evidence invites us to reconsider the established sequence of events in this type of patient. However use of two biologics in one patient is currently prohibitively expensive.

## Latest techniques

Hereditary haemorrhagic telangiectasia (HHT) is a genetic condition characterized by multiple arteriovenous malformations (AVMs) in which there are direct connections between arteries and veins, without intervening capillaries. Spontaneous epistaxis, beginning around 12 years of age, are the most common clinical feature and are difficult to treat (8). Kumar et al. conducted a comparative analysis of the therapeutic efficacy of intralesional sclerotherapy employing 3% sodium tetradecyl sulfate, in contrast to non-sclerotherapy-based treatment modalities, for the management of epistaxis associated with Hereditary Haemorrhagic Telangiectasia (HHT). This retrospective study demonstrated that, in comparison with non-sclerotherapy treatments, intralesional sclerotherapy for epistaxis in HHT is more efficacious in reducing breakthrough epistaxis and exhibits reduced intraoperative blood loss.

## Conclusion

The articles included in this Research Topic collectively reinforce the need for clinically actionable, patient-centred approaches to upper-airway disease. Advancements in research, definitions, diagnostic frameworks, and therapeutic strategies underscore the growing emphasis on personalised management. The emerging evidence on environmental influences, inflammatory mechanisms, and multimorbidity further underscores the importance of early recognition, appropriate referral pathways, and integrated airway care. Collectively, these contributions provide practical insights that can assist clinicians in optimising outcomes for patients with allergic rhinitis, chronic rhinosinusitis, and related airway disorders.
